# Contrast-enhanced ultrasound: clinical applications in patients with atherosclerosis

**DOI:** 10.1007/s10554-015-0713-z

**Published:** 2015-07-24

**Authors:** Arend F. L. Schinkel, Mathias Kaspar, Daniel Staub

**Affiliations:** Department of Cardiology, Thoraxcenter, Erasmus MC, Rotterdam, The Netherlands; Division of Angiology, Department of Internal Medicine, University Hospital Basel, Basel, Switzerland

**Keywords:** Aortic aneurysm, Atherosclerosis, Carotid artery, Contrast-enhanced ultrasound, Echocardiography, Endoleak, Endovascular aneurysm repair, Intima media thickness, Molecular imaging, Myocardial perfusion imaging, Thrombus

## Abstract

Contrast-enhanced ultrasound (CEUS) is increasingly being used to evaluate patients with known or suspected atherosclerosis. The administration of a microbubble contrast agent in conjunction with ultrasound results in an improved image quality and provides information that cannot be assessed with standard B-mode ultrasound. CEUS is a high-resolution, noninvasive imaging modality, which is safe and may benefit patients with coronary, carotid, or aortic atherosclerosis. CEUS allows a reliable assessment of endocardial borders, left ventricular function, intracardiac thrombus and myocardial perfusion. CEUS results in an improved detection of carotid atherosclerosis, and allows assessment of high-risk plaque characteristics including intraplaque vascularization, and ulceration. CEUS provides real-time bedside information in patients with a suspected or known abdominal aortic aneurysm or aortic dissection. The absence of ionizing radiation and safety of the contrast agent allow repetitive imaging which is particularly useful in the follow-up of patients after endovascular aneurysm repair. New developments in CEUS-based molecular imaging will improve the understanding of the pathophysiology of atherosclerosis and may in the future allow to image and directly treat cardiovascular diseases (theragnostic CEUS). Familiarity with the strengths and limitations of CEUS may have a major impact on the management of patients with atherosclerosis.

## Introduction

Contrast-enhanced ultrasound (CEUS) is an increasingly used imaging modality in cardiovascular medicine, and has advantages for both cardiac and vascular imaging [1–3]. CEUS is characterized by the use of an ultrasound contrast agent in conjunction with ultrasound imaging. The ultrasound contrast agent consists of gas filled microbubbles, which resonate when exposed to an ultrasound beam. CEUS can be used to improve the image quality of standard ultrasound or to obtain information that cannot be assessed using standard ultrasound [4, 5].

CEUS has various potential applications in patients with atherosclerosis (Table 1). In those with known or suspected coronary artery disease, CEUS improves the delineation of endocardial borders, allowing an accurate assessment of left ventricular shape and function. The use of an ultrasound contrast also improves the image quality and diagnostic accuracy of stress echocardiography. In patients with a previous myocardial infarction, CEUS is useful to assess intracardiac thrombus, which may have important clinical implications.

**Table 1:** Overview of clinical application of contrast-enhanced ultrasound in cardiovascular diseases

Field of application	Clinical indication
Cardiac	Endoluminal border delineation to assess left ventricular volumes and function and detection of intracardiac thrombus
	Visualization of wall motion and thickening to assess myocardial ischemia and viability
	Quantification of myocardial perfusion
Carotis	Endoluminal border delineation to depict hypoechogenic plaques, plaque irregularities and ulcerations, distinguish very high-grade stenosis from complete occlusion
	Detection and quantification of intraplaque neovascularization to risk stratify atherosclerotic lesions and to monitor therapeutic effects
Aorta	Detection of dissection membrane and re-entry and discrimination of true and false lumen in abdominal aortic dissection
	Endoluminal border delineation in abdominal aortic aneurysm to detect intraluminal thrombus
	Detection and classification of endoleaks after endovascular aortic aneurysm repair (EVAR)

In patients with known or suspected carotid atherosclerosis, CEUS can be used to assess the presence and extent of atherosclerosis. Moreover, CEUS allows to characterize the atherosclerotic plaque and evaluate factors that are associated with plaque rupture, including assessment of plaque surface, plaque ulceration and intraplaque vascularization. Furthermore, CEUS imaging can further increase the diagnostic performance in different aortic pathologies, particularly the detection and characterization of endoleaks following endovascular treatment of abdominal aortic aneurysms (AAAs).

This review of literature will explain the principles and ultrasound acquisition settings, and will focus on cardiac and vascular including carotid and aortic applications of CEUS.

### Principles and settings of CEUS

#### Ultrasound contrast agents

A number of ultrasound contrast agents has been developed and is commercially available. The ultrasound contrast agent typically consists of microbubbles with a protein or lipid shell filled with an inert gas. These microbubble contrast agents are stable, and are strong reflectors and resonators when exposed to an ultrasound beam. An overview of commercially available contrast agents is provided in Table 2. The safety of intravenous administration of an ultrasound contrast agent has been confirmed in millions of patients [6, 7]. Contraindications for the administration of the contrast agents are: known allergy to the contrast agent, large right to left shunt, and an unstable clinical condition. It is recommended to use a protocol in the echo laboratory for early recognition of side-effects and so that in the event of an allergic reaction immediate treatment can be started [8].

**Table 2:** Overview of commercially available ultrasound contrast agents

Contrast agent	Manufacturer	Shell	Gas
Definity	Lantheus medical imaging	Lipid	Octafluoropropane
Levovist	Schering AG	Galactose	Air
Optison	GE healthcare	Albumin	Octafluoropropane
SonoVue	Bracco diagnostics	Lipid	Sulfurhexafluoride
Sonazoid	GE healthcare	Lipid	Perfluorocarbon

#### Ultrasound system settings

The currently available high-end ultrasound systems have preprogrammed settings for CEUS. These settings can be slightly adapted to optimize the CEUS study. To avoid destruction of the microbubbles, which are fragile, a low mechanical index (0.1–0.3) or middle-high (0.3–0.5) mechanical index is selected [8]. A low mechanical index allows continuous image acquisition, whereas a middle-high mechanical index requires intermittent imaging (for example acquisition of 1 frame every 2 or 3 cardiac cycles) allowing the replenishment of destructed microbubbles. Depending on the ultrasound system settings, harmonic imaging is used which is based on differences in ultrasound reflection by tissue and by the contrast agent. The contrast agent does not only reflect the ultrasound at the transmitted frequency but also at higher harmonic frequencies, allowing to distinguish contrast agent from tissue. Other contrast-specific ultrasound methods are based on the transmission of multiple ultrasound pulses. Mostly, a combination of pulses is transmitted which are out-of-phase (pulse inversion), or differ in amplitude (power modulation), depending on the manufacturer of the ultrasound system.

#### CEUS acquisition

After explanation of the imaging modality and obtaining informed consent of the patient, the CEUS examination can be started. First, a venous infusion line is placed, and after preparation of the contrast agent according to the instructions of the manufacturer, the ultrasound contrast agent is administered intravenously. The ultrasound contrast agent can be injected as a bolus which is practical and will give good imaging results in most circumstances. Alternatively, the ultrasound contrast agent can be administered using a continuous infusion, which provides a stable concentration of contrast agent in the circulation and therefore has advantages for the assessment of myocardial perfusion. After intravenous administration, the ultrasound contrast agent travels through the cardiovascular system. The microbubbles behave as red blood cells and are strict intravascular tracers. Because of their small diameter, the microbubbles are able to pass the pulmonary circulation. After administration, the contrast agent can be visualized for minutes. When the microbubbles are shattered, the shell is removed through the reticuloendothelial system, while the inert gas is exhaled.

## CEUS: cardiac applications

### Endocardial border delineation

#### Assessment of left ventricular volumes and function

An accurate detection of the endocardial border is highly relevant in patients with known or suspected coronary artery disease. Up to 15 % of these patients have a moderate to poor image quality at standard echocardiography, because of comorbidity such as obesity and chronic pulmonary disease. CEUS significantly improves the detection of the endocardial border, which is clinically relevant for an accurate assessment of left ventricular volumes and systolic left ventricular function [9]. The use of an ultrasound contrast agent improves the assessment of the left ventricular ejection fraction, and thereby has an impact on clinical care [10]. Among other parameters, the left ventricular ejection fraction determines the medical treatment, therapy with an implantable cardioverter defibrillator and finally the prognosis of the patients.

#### Contrast stress echocardiography

Stress echocardiography provides information on the presence and extent of myocardial ischemia. Moreover, stress echocardiography allows assessment of myocardial viability in patients with ischemic cardiomyopathy. A good visualization of wall motion and thickening is needed for an accurate interpretation of stress echocardiography. Because the criteria for myocardial ischemia and myocardial viability are based on wall motion abnormalities, an accurate visualization of all walls of the left ventricle is required. Ultrasound contrast agents provide an improved endocardial border delineation, and result in a better reproducibility in wall motion analysis even by less experienced readers [11, 12]. The use of an ultrasound contrast agent is recommended during stress echocardiography in all patients with suboptimal image quality, in whom ≥ 2 segments of the left ventricle are not adequately visualized. The administration of an ultrasound contrast agent is particularly relevant for visualization of the anterior and lateral wall, because image quality is often suboptimal in those areas.

#### Intracardiac thrombus

**Fig. 1:**
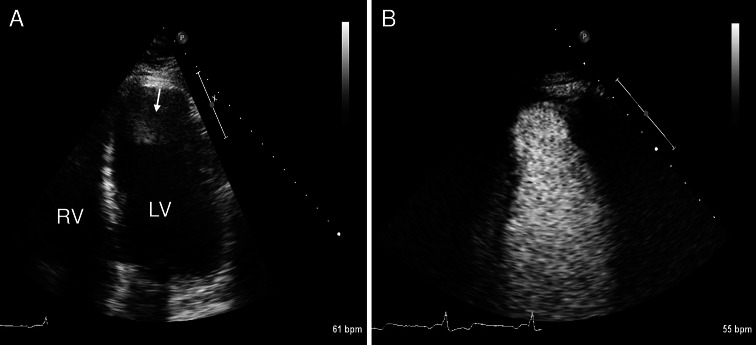
Assessment of an intracardiac thrombus using CEUS. A 61-year old man with a history of a large apical-anterior myocardial infarction was referred for echocardiography for the evaluation of cardiac thrombus. **a** Standard echocardiography (apical 4 chamber view) demonstrates an abnormality in the left ventricular apex which was a suspected thrombus (*arrow*). **b** CEUS demonstrates that there is actually no thrombus in the left ventricular apex. The abnormality that was observed on standard echocardiography was probably a reverberation artefact

In patients with ischemic cardiomyopathy and a suspected intracardiac thrombus, CEUS may be highly useful. In these patients the endocardial border in the left ventricular apex is frequently difficult to delineate, and clutter or reverberation artefacts near the apex may be present during standard echocardiography. CEUS allows a reliable assessment of the left ventricular cavity and can be used to exclude or confirm the presence of an intracardiac thrombus (Fig. 1). A study in 409 patients demonstrated that standard echocardiography was nondiagnostic for the exclusion or detection of thrombus in 46 % of the cases; a selection of these patients subsequently underwent CEUS, and the addition of an ultrasound contrast agent led to a diagnostic study in 90 % of the cases [13]. Recent multimodality imaging studies have confirmed that CEUS has a higher diagnostic accuracy compared to standard echocardiography for the assessment of intracardiac thrombus, and demonstrated that contrast-enhanced magnetic resonance imaging may be even superior [14, 15].

The evaluation of cardiac thrombus by CEUS may have a direct impact on the management of the patient, and may lead to changes in medical therapy (anticoagulation). Additionally the detection of an intracardiac thrombus by CEUS may prevent defibrillation threshold testing in patients with an implantable cardioverter defibrillator (ICD).

### Myocardial perfusion imaging

**Fig. 2:**
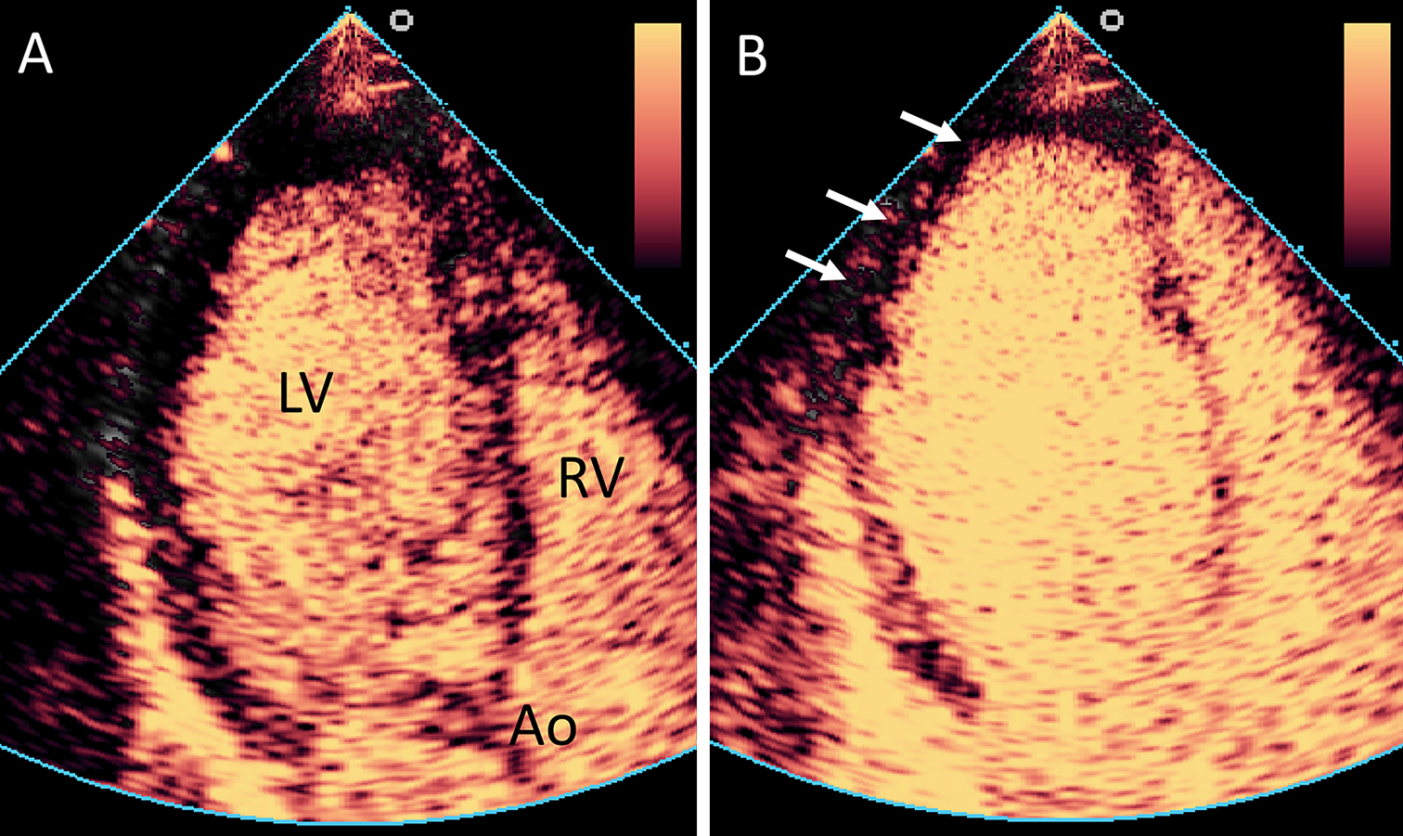
Assessment of myocardial perfusion using CEUS. Example of an abnormal myocardial perfusion echocardiogram. **a** Apical three chamber view. After administration of the contrast agent, a high mechanical index flash is given to destroy the contrast agent that is present in the myocardium. Thereafter, the left ventricular myocardium does contain no or only a limited amount of contrast agent. *Ao* aorta, *LV* left ventricle, *RV* right ventricle. **b** After a short period, the myocardium is filled with blood and contrast agent. There is an apical and lateral perfusion defect visible (*arrows*), indicating a significant coronary stenosis. Example reproduced from [84]

In patients with known or suspected coronary artery disease, CEUS may be useful for the assessment of myocardial perfusion (Fig. 2) [16]. Myocardial perfusion imaging requires a stable concentration of the ultrasound contrast agent in the circulation, that can be best achieved with intravenous administration using a continuous pump infusion system. After a high mechanical index ultrasound flash to destruct all intramyocardial ultrasound contrast agent, the replenishment of the contrast agent into the myocardium is recorded. Several software packages were developed to measure the video-intensity of the myocardial perfusion replenishment and to quantify myocardial perfusion defects. Although CEUS myocardial perfusion imaging has been successfully used in multiple studies [16, 17], the use of this imaging modality for this specific application clinical practice is still limited by a relatively high intra- and interobserver variability.

### CEUS: carotid applications

#### Standard carotid ultrasound

In daily routine, the use of standard carotid ultrasound to detect atherosclerotic wall alterations including carotid plaques and stenosis is well established. The main focus of such an investigation is the measurement of Doppler velocities within carotid lesions in order to determine the grade and therefore also the severity of the stenosis. Beside this morphological and hemodynamic information on conventional ultrasound which represent s a late manifestation of atherosclerosis, also early changes of the atherosclerotic process can be depicted by using high frequency B-mode ultrasound. It is well known that small changes in thickening of the carotid intima-media (c-IMT) can be detected using this imaging method, and represent an early surrogate marker of systemic atherosclerosis [18]. Furthermore, B-mode ultrasound can also be useful not only to detect but also to characterize atherosclerotic plaque by identifying surface irregularities and echogenicity of the lesion itself. In large prospective epidemiological studies, it has been shown that an increased c-IMT is associated with future cerebrovascular and cardiac events [19]. However, in addition to the traditional cardiovascular risk factors to predict individuals risk, the measurement of c-IMT has limited value [20]. On the other hand, the combination of c-IMT and the presence of carotid plaque has the potential to significantly increase risk prediction in addition to traditional cardiovascular risk factors alone [21].

Nevertheless, it is still controversial if the incorporation of c-IMT and carotid plaque to cardiovascular risk assessment strategies is really beneficial in the clinical setting [22]. Therefore, it seems to be useful to include further plaque characteristics which can be analyzed by ultrasound imaging for a better risk stratification of individual patients [23]. Based on different studies, patients with hypoechoic carotid lesions and plaque ulcerations on B-mode ultrasound have more cerebrovascular and cardiac events in the future, and therefore, this kind of alterations are associated with higher cardiovascular risk [3, 24–27].

#### CEUS for luminal enhancement of the carotid artery

**Fig. 3:**
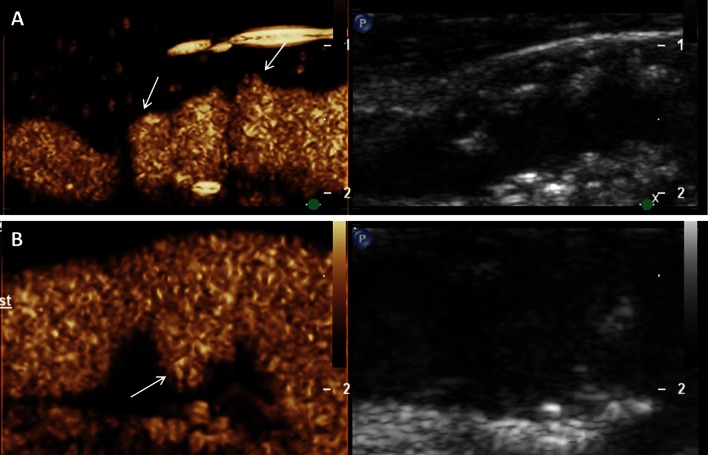
Assessment of vessel wall irregularities and plaque ulcerations on carotid artery using CEUS. **a** Mixed hypo- and hyperechoic plaques at the carotid bulb on B-mode ultrasound (*right side*) and CEUS imaging (*left side*) with surface irregularities (*arrows*). **b** Plaque ulceration (*arrow*) on CEUS imaging (*left side*) at the origin of the internal carotid artery not detected on B-mode ultrasound (*right side*)

During the last years CEUS imaging of the carotid artery has been widely investigated in order to analyze carotid plaque characteristics in more detail compared to standard ultrasound alone. Usually, a linear vascular ultrasound probe with medium frequency (e.g. 3–9 MHz) is most suitable for carotid artery imaging with CEUS. Typically, on CEUS imaging the carotid lumen is enhanced shortly after injection of the contrast agent. The adventitia layer also appears enhanced, whereas the intima-media layer remains hypoechogenic. Using this imaging technique, smaller vessel wall irregularities and hypodense plaques, as well as plaque ulcerations can be depicted much better than using standard ultrasound alone [28] (Fig. 3).

Recently, asymptomatic patients with high cardiovascular risk have been investigated using carotid CEUS imaging in order to detect subclincal atherosclerotic lesions [29]. The researchers found that this additional use of CEUS increases the detection rate of predominantly hypoechogenic carotid plaque. Therefore, carotid CEUS may be useful to evaluate patients for subclinical atherosclerosis. Furthermore, different reports also emphasize that CEUS imaging could be very useful to separate a carotid occlusion from a very narrow stenosis [30, 31]. Carotid CEUS imaging could also be very beneficial for analyzing restenosis after carotid stenting [32].

#### CEUS for carotid plaque neovascularization

Carotid CEUS imaging is not only useful for better delineation of the endoluminal border of the carotid artery but also for a deeper analysis of plaque characteristics by visualizing the microvessels within the atherosclerotic lesion itself [2]. These microvessels have been known to be derived from the physiological existing vasa vasorum in the adventitia layer of the large- and middle-size arteries which can proliferate into the atherosclerotic plaque. In different histological studies, the increase of intraplaque neovascularization has been investigated and seems to be triggered by hypoxia and inflammation. Importantly, plaque progression and vulnerability, eventually leading to the vascular event seems to be closely related with this intraplaque neovascularization [33]. Particularly in symptomatic carotid stenosis, such microvessels of larger amount have been documented. Interestingly, this network of small vessels seems of immature nature and leakier than normal microvessels which make such plaque more prone for inflammatory cell recruitment and intraplaque hemorrhage, eventually leading to plaque rupture and vascular event. Hellings and co-workers published an important prospective study in which specific histological characteristics of carotid stonsis after carotid endarterectomy were analyzed with regard to future vascular events [34]. Patients with higher vessel density and more intraplaque hemorrhage on histology had during the 3 years follow-up more vascular events. These results emphasize again that higher neovascularization within the carotid atherosclerotic plaque seems to be a marker of cardiovascular vulnerability with a certain prognostic importance. Therefore, imaging modalities which help to visualize intraplaque neovascularization non-invasively could be very useful for further stratification and prevention of cardiovascular risk [2, 35].

**Fig. 4:**
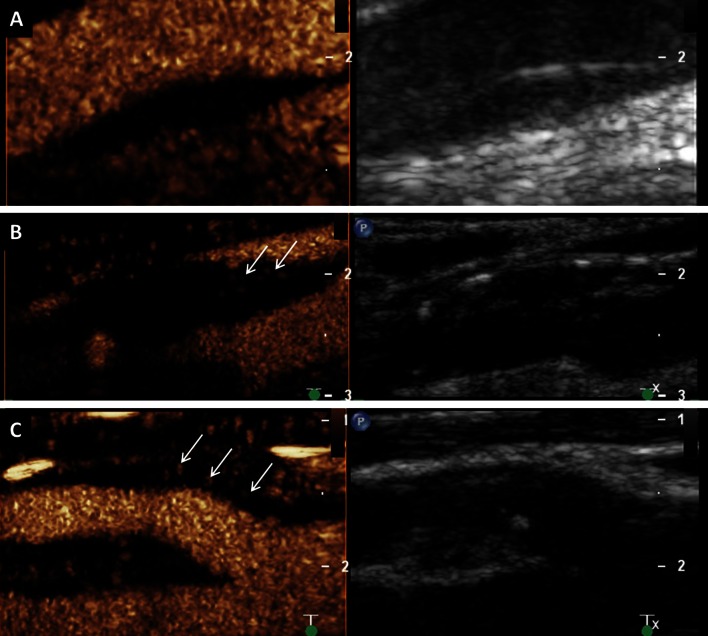
Visual based grading of intraplaque neovascularization on CEUS imaging. **a** No enhancement: Small plaque on the fare wall of the internal carotid artery on B-mode ultrasound (*right side*) without intraplaque neovascularization on CEUS imaging (*left side*). **b** Moderate enhancement: Mixed hypo- and hyperechoic plaques at the carotid bulb on B-mode ultrasound (*right side*) and CEUS imaging (*left side*) with moderate intraplaque neovascularization on the plaque shoulder (*arrows*). **c** Extensive enhancement: Hypoechoic plaque at the origin of the internal carotid artery on B-mode ultrasound (*right side*) and CEUS imaging (*left side*) with extensive intraplaque neovascularization including the plaque core (*arrows*)

Particularly, carotid CEUS imaging seems to be of great value for the identification and quantification of such microvessels within carotid arteriosclerotic lesions. Several researchers analyzed this intraplaque neovascularization on CEUS imaging in different animal models [36–38] and also in patients scheduled for carotid endarterectomy [39, 40]. They found good correlations between the grade of intraplaque neovascularization on CEUS and the amount of microvessels on histology. Usually, the degree of intraplaque neovascularization on CEUS was determined based on a visual interpretation using a scoring system for the grading (e.g. no, moderate, extensive enhancement) (Fig. 4). Some investigators tried to quantify the degree of intraplaque neovascularization by measuring video-intensity within the atherosclerotic lesion [41–43]. They also documented a very good correlation between this quantitative approach to measure the degree of intraplaque neovascularization and microvessel density on histology.

Recently, van den Oord published the results of a new quantification tool based on custom developed software which uses a motion tracking algorithm [44]. Such a more accurate quantitative analysis method to assess intraplaque neovascularization seems to be mandatory for clinical application of CEUS imaging in the future. Interestingly, the researchers found also a good correlation between the degree of intraplaque neovascularization using this quantification tool and the previous mentioned visual based approach on carotid CEUS imaging. This assessment seems also to be very reproducible with low intra-observer and inter-observer variability. Similar to this study, other studies used also a quantitative software based approach to determine the degree of intraplaque neovascularization on carotid CEUS imaging [45, 46]. They found a good correlation with histological analysis of plaque vascularization, too.

**Fig. 5:**
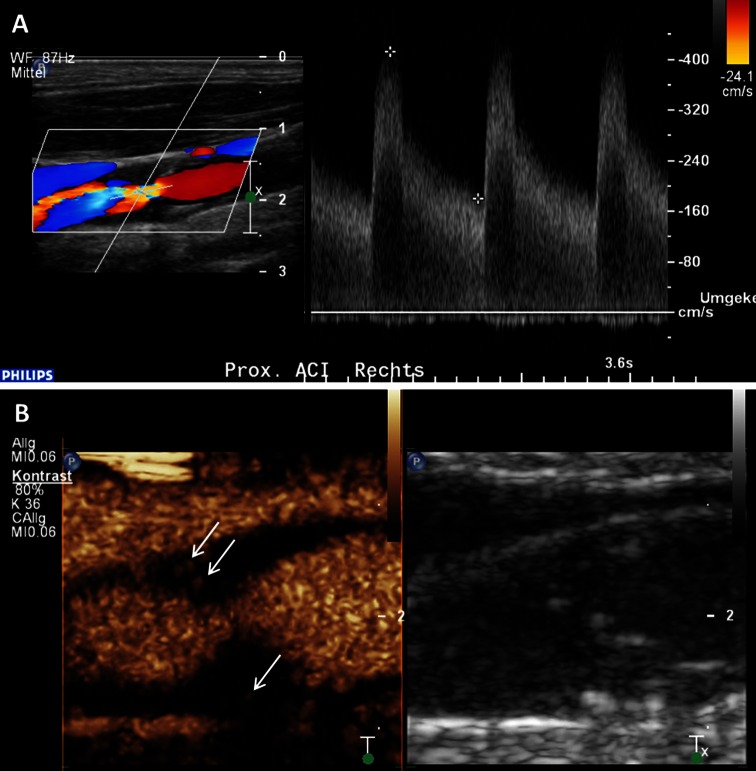
Intraplaque neovascularization within carotid stenosis on CEUS imaging. **a** 77-year-old patient with asymptomatic high-grade stenosis at the origin of the right internal carotid artery on Duplex ultrasound. **b** Extensive intraplaque neovascularization within the carotid stenosis at the near wall on CEUS imaging (*arrows*) and moderate neovascularization at the fare wall (*arrow*)

Our research group also analyzed almost 300 atherosclerotic carotid lesions with standard ultrasound and CEUS imaging [47]. We measured intraplaque neovascularization on CEUS using the previously mentioned visual based scoring system (no, moderate or extensive enhancement). In line with previous published results and with the concept that hypoechoic plaques were more vulnerable, we found that echogenicity on B-mode ultrasound was inversely correlated with the degree of intraplaque neovascularization on CEUS. Hypoechoic plaques were significantly more often more vascularized on CEUS imaging. We also revealed that more severe atherosclerotic lesions based on the degree of stenosis and plaque thickness were also more vascularized on CEUS imaging (Fig. 5). One recent study also documented that neovascularization within carotid stenosis detected by CEUS was associated with the presence of microembolic signals known as another marker of plaque vulnerability using transcranial color Doppler monitoring [48].

Furthermore, clinical vascular symptoms have been correlated with the presence of intraplaque neovascularization on CEUS. Retrospective studies including our own have shown pronounced intraplaque neovascularization on carotid CEUS imaging in patient with past cerebrovascular and coronary events [43, 49–52]. This result supports the concept that the vascular risk assessment based on the carotid vessel wall alterations, particularly the degree of intraplaque neovascularization is not limited to the cerebrovascular bed but also generalizable to the coronary and maybe also the peripheral vascular system.

The use of carotid CEUS imaging to assess intraplaque neovascularization in order to have a new, non-invasive tool for better risk stratify carotid lesions and patients has been recommended by the latest EFSUMB guidelines on the clinical practice of CEUS on non-hepatic applications [53]. Even though there are no clinical date, the guidelines also emphasize the benefit of carotid CEUS imaging also for the assessing the response to anti-atherosclerotic therapy. Interestingly, recent published paper demonstrated in rat model, that atorvastatin significantly inhibits the development of adventitial vasa vasorum and progression of atherosclerosis measured by CEUS and intravascular ultrasound imaging independent of lowering the cholesterol level [54]. Therefore, monitoring of atherosclerotic lesions by CEUS imaging could indeed be clinically beneficial in the future.

Actual good clinical indication for CEUS imaging is the detection of small hypoechoic and ulcerated plaque by a better delineation of the endovascular border based on the luminal enhancement. Furthermore, CEUS imaging for the quantification of intraplaque neovascularization seems to be a good tool for better risk stratification of atherosclerotic carotid stenosis and patients with carotid plaques. This strategy could improve the prediction of future vascular events and may be helpful for better treatment selection. Particularly, in patients with asymptomatic carotid stenosis, CEUS imaging could be beneficial to select those patients who should assign for carotid endarterectomy or stenting. However, further prospective randomized studies to analyze this approach are mandatory before incorporating such a concept in a daily clinical algorithm.

### CEUS: aortic applications

Also the use of standard ultrasound in the diagnostic approach of the abdominal aorta is well established. In recent years the use of CEUS increasingly allowed a more differentiated view of the aortic wall by a better demarcation of the aortic lumen and its branches. In the following part we will discuss the main implications of CEUS in imaging different atherosclerotic pathologies of the abdominal aorta.

#### Aneurysm dissecans

The clinical presentation of a dissection of the abdominal aorta varies widely and the prediction of the progression of a dissection is challenging, and therefore more than one-third of aortic dissections remain initially undetected and nearly 30 % are diagnosed post mortem by autopsy [55–57].

Due to its high spatial resolution and its high rating in routing therapy the definite diagnosis or the exclusion of an aortic dissection is made by computed tomography angiography (CTA). Conventional ultrasound is helpful in early stages of finding the diagnose in an emergency set-up with suspected dissection of the abdominal aorta, whereas the addition of contrast agent leads to a better differentiation of true and false lumen, because the latter, if not full of thrombus, usually shows contrast flow during late phase or at least with noticeable detention [58]. Clevert et al. [59] evaluated 35 patients with abdominal aortic dissection using standard ultrasound, CEUS imaging and CTA. They found that sensitivity of CEUS imaging to detect dissection membrane was 97 % using CTA as gold standard which was much better than standard ultrasound alone (sensitivity 68 %). Particullarly, in search of the entry or re-entry of the dissection, CEUS can be used, especially to detect small dissection membranes, which were not able to be visualized with B-Mode or color Doppler.

#### Aneurysm verum

Over the last decade standard ultrasound was not only entrenched as a screening method for AAA but also as a valid method during its routine follow-up [57]. Different clinical reports have showed the importance of CEUS in imaging the aneurysm sack with its perfused lumen and the distinction of thrombotic structures [56, 58]. Interestingly, even in the setting of ruptured aortic aneurysm, it seems, that collecting contrast-specific images such as enhancement of the aortic wall or contrast containing extravasates preoperatively does not delay surgery [60].

#### Endovascular aortic aneurysm repair (EVAR) and Endoleak

Catheter based endovascular aneurysm repair (EVAR) is meanwhile a worldwide established alternative to conventional open surgery replacement in treating an aortic aneurysm and provides a minimal invasive option especially in patients with fitting characteristics of the aorta [61].

**Fig. 6:**
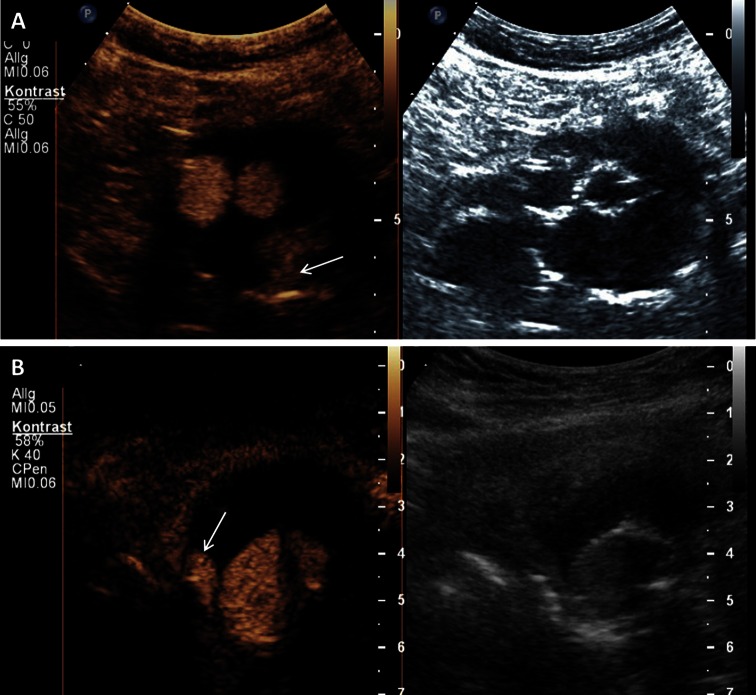
Endoleak after endovascular aortic aneurysm repair on CEUS imaging. **a** Typ 2 endoleak with enhancement of the aneurysm sac (*arrow*) caused by retrograde flow form a lumbar artery on CEUS imaging (*left side*) and corresponding B-mode ultrasound (*right side*). **b** Typ 1 endoleak with enhancement of the aneurysm sac (*arrow*) by an incomplete seal at the proximal end of the graft (main body) on CEUS imaging (*left side*) and corresponding B-mode ultrasound (*right side*)

However, EVAR shows to a certain extent complications that are procedure associated and gain in relevance especially with prolonged survival. These are predominantly so called endoleaks which occur in up to 45 % of the cases and are characterized by a persistent blood flow into the aneurysm sac from outside the endoprothesis [62]. Here, a progressive aneurysmal enlargement by flow-induced pressure increase could lead to a relocation of the stent graft or in the further course even to the rupture of the aneurysm sac. Therefore, the detection of such an enlargement of the aneurysm sac makes a reintervention often necessary. Nowadays, complication controls are often made by contrast CTA [63]. However, because of the radiation burden as well as the potentially nephrotoxic effect of the contrast medium with frequent use, the role of routine follow-up of patients using CTA is controversial [64]. The long term prognosis after EVAR is strongly dependent on the renal function and could be compromised by repetitive application of CT-specific contrast agents, and therefore CEUS appears to be an enticing option [65]. CEUS allows the real-time assessment of flow, which is highly useful for the detection and classification of endoleaks (Fig. 6). A meta-analysis showed an accumulated specificity of 98 %, respectively a sensitivity of 88 %, for the finding of an endoleak and the authors stated a superiority of CEUS in comparison with standard ultrasound, which is congruent with previously collected data [65, 66].

Due to the strictly intravascular distribution and resonance pattern of the contrast agent, CEUS can also be used for detection of endoleaks that are difficult or even impossible to be displayed by CTA due to low flow rates. In one study, CEUS was used additionally in a small number of patients that did not show any signs of endoleak or endograft irregularities during CTA despite increase in aneurysm diameter after EVAR and surprisingly revealed in 100 % of the participants an endoleak and helped to classify it correctly in 80 % of the cases. Results were confirmed by a final subtraction angiography and led to the conclusion that in the event of an unclear aneurysm enlargement after EVAR, CEUS represents a promising diagnostic tool [67]. In addition, CEUS can also be used intraoperatively for early detection of endoleaks or to find appropriate landing zones during EVAR and so important information can be collected to carry out immediately a correction of the stent position during the intervention [68, 69].

Another application in the field of CEUS for endoleak detection, especially to differentiate those with variable flow rates, is the by contrast harmonic imaging optimized perfusion analysis [70]. Hereby, reperfusion of abdominal aneurysm sac after EVAR is determined by a time intensity curve, which, in turn, is derived from bolus administration of contrast agent.

#### CEUS for post-interventional follow-up after EVAR

In general, CEUS is at least equated to CTA in the diagnostic performance in terms of recognition and classification of endoleaks. This led to the conclusion of some authors that for future references CEUS might play a deciding role in post-interventional follow-up after EVAR [71]. In a prospective observational study of more than 100 patients after EVAR to examine the accuracy rate of various diagnostic procedures compared to conventional angiography CEUS proved as superior against color Doppler and as equivalent against CTA or MRA [72]. Moreover, the authors even conclusively stated a superiority of CEUS in comparison to CTA with respect to the classification of endoleaks. This is in turn connected to another smaller analysis, in which patients after EVAR were examined over a longer period of time on endoleaks, whereby even insidious ones or those with low flow could be visualized. However, due to continuous administration of contrast medium here instead of basal-bolus principle the examination window has been extended in time, thus allowing a more precise consideration [73]. The accuracy of CEUS in the recognition and classification of endoleaks after EVAR seems to be high, as recently demonstrated prospectively with a sensitivity of 97 %, a specificity of 100 % and an accuracy of 99 % [74].

#### 3D-CEUS for endoleak detection

As a further development of CEUS, a novel technique for three-dimensional CEUS (3D-CEUS) utilizes positional information from magnetic field emitters to assemble all ultrasonic reflections into a high-definition three-dimensional image [75]. Ormesher et al. [76] stated, that in patients undergoing conventional infrarenal EVAR electively this 3D-CEUS technique allows intraoperatively the detection of endoleaks not seen on unipolar digital subtraction angiography and is more sensitive in finding the source of endoleak than conventional CEUS. This led to the authors implication that 3D-CEUS has the potential to complement or even to replace digital subtraction angiography in this context as final imaging in reduction of x-ray contrast. In another study, which was conducted by the same research group, the authors conclude that 3D may be more sensitive in assessing an endoleaks after EVAR than 2D CEUS or CTA [75].

Hopes for the future are through the use of CEUS peri-interventionally during EVAR to improve risk stratification with respect to the occurrence of complications, so that therapy management or the follow-up intervals can be individually customized.

### Future directions in cardiovascular CEUS

Ultrasound imaging using microbubbles which are targeted with monoclonal antibodies to specific ligands could further improve and expand the diagnostic prospects of current cardiovascular ultrasound examination in the future. The use of such targeted microbubbles may allow non-invasively investigating specific molecular processes that play a role in the pathophysiology of cardiovascular diseases [77].

In atherosclerosis, targeted microbubbles have been examined in the assessment of thrombosis, neoangiogenesis and inflammation in various animal models. Wang and co-workers investigated CEUS imaging with glycoprotein IIb/IIIa-targeted microbubbles which bind specifically to activated platelets [78]. They imaged the carotid artery in a mice model after thrombus induction and after thrombolysis. This imaging method allowed real-time molecular imaging of acute arterial thrombosis and monitoring of the success or failure of pharmacological thrombolysis in vivo. In a mouse model of age-dependent atherosclerosis, ultrasound molecular imaging of the proximal thoracic aorta was performed with microbubbles targeted to P-selectin and VCAM-1 in order to detect a lesion-prone vascular phenotype [79]. Both, P-selectin and VCAM-1 are involved in the regulation of leukocyte trafficking. This is an early step in inflammatory process involved in plaque formation. The researchers found that this targeted microbubbles preferentially bind to regions of lesion formation. Using this same mouse model, targeted microbubbles to VCAM-1 for CEUS imaging was used to investigate also the effect of statins to this early atherosclerosis process [80]. Less endothelial expression of VCAM-1 and reduced plaque burden was found in statin treated animals. Accordingly, signal enhancement by CEUS molecular imaging was detected only in non-treated, but not in statin-treated animals. Monitoring these early changes of an activated and inflamed endothelium during the atherosclerotic process is appealing and has already made its way to preclinical studies in non-human primates [81]. Molecular ultrasound imaging has also been used to investigate a later stage of atherosclerotic disease by using VEGF-receptor targeted microbubbles in order to detect neovascularization on the abdominal artery plaques in rabbits [82]. This could be helpful to better risk stratify atherosclerotic lesions by imaging more specifically vulnerable plaques. However, no clinical studies using molecular ultrasound imaging in cardiovascular disease have been performed so far. Therefore, further studies are needed to bring the targeted microbubbles technology successfully forward from the laboratory to the clinical setting.

Moreover, ultrasound contrast agent has the potential to even further increase not only the diagnostic but also the therapeutic capabilities of ultrasound technology in the cardiovascular field. Several researchers are already investigating ultrasound directed and site-specific gene and drug delivery systems [83]. Particularly, the use of small molecules or plasmid DNA for thrombolysis, anti-inflammatory or anti- or angiogenic treatment could have an important clinical impact. Eventually, these newer techniques of theragnostic CEUS with the possibility to improve diagnostic imaging and directly treat the patient could be of great clinical benefit in the field of atherosclerosis.
